# Leadership, cohesion, and stress in primary care facilities and retention in chronic care in rural northeast South Africa before and during the COVID-19 pandemic: A longitudinal study

**DOI:** 10.7189/jogh.14.05035

**Published:** 2024-12-09

**Authors:** Hannah H Leslie, Morelearnings Sibanda, Kathleen Kahn, Stephen M Tollman, Nkosinathi Masilela, F Xavier Gómez-Olivé, Sheri A Lippman, Chodziwadziwa W Kabudula

**Affiliations:** 1 Division of Prevention Science, Department of Medicine, University of California, San Francisco, San Francisco, California, USA; 2 MRC/Wits Rural Public Health and Health Transitions Unit, School of Public Health, University of the Witwatersrand, Johannesburg, South Africa

## Abstract

**Background:**

Human immunodeficiency virus (HIV) and hypertension are major contributors to morbidity and mortality in South Africa. Effective management of these conditions is critical to population health, yet patient management and retention varies by facility for reasons that are not fully understood. We assessed whether measures of clinic leadership, cohesion, and stress were associated with retention for HIV and hypertension in a cohort of patients in northeast South Africa before and during the Coronavirus disease 2019 pandemic.

**Methods:**

We quantified nursing capacity and service readiness within primary health care facilities in the Bushbuckridge sub-district in Mpumalanga province South Africa. We administered brief scales on facility leadership, cohesion, and stress from January to March 2019, and tested scales for individual and facility-level agreement. We extracted clinical records for patients with HIV and/or hypertension from 2019 to 2021 and quantified treatment retention by quarter. We used generalised estimating equations to assess individual and clinic factors associated with retention in each treatment programme prior to (2019–first quarter 2020) and during (second quarter 2020–2021) the pandemic.

**Results:**

The nine facilities had a median of 12 nurses on staff and scored 0.83 out of 1.0 on basic service readiness. We collected responses to leadership, cohesion, and stress scales from 54 nurses and counsellors. Scales showed high inter-item agreement and moderate within-facility agreement. From 2019 to 2021, 19 445 individuals were treated for HIV and/or hypertension across seven participating facilities. Two-year retention was 91% for those with both conditions, 82% for those in treatment for HIV alone and 77% for those in treatment for hypertension alone, with 10–15% differences between facilities and high retention during the pandemic period. In addition to those with both conditions, women and adults aged 60–69 were more likely to be retained. Clinic factors were inconsistently associated with patient retention.

**Conclusions:**

While measures of clinic leadership, cohesion, and stress were generally reliable at individual and facility levels, we found limited evidence supporting a link between these factors and better retention in care. Retention was stable during the Coronavirus disease 2019 pandemic. Men, the youngest and oldest adults, and those without known multimorbidity should be prioritised for retention interventions.

South Africa currently has an estimated 7.8 million people living with human immunodeficiency virus (HIV) and 5.6 million on Antiretroviral therapy (ART) [1], including older adults increasingly at risk for non-communicable diseases (NCDs) [2,3]. Cerebrovascular and heart diseases joined HIV in the top six underlying causes of death in South Africa in 2018, accounting together for nearly 20% of all deaths in the country [4]. Hypertension is one of the most important risk factors for cerebrovascular and cardiac mortality and one of the most responsive to intervention [5,6]. Hypertension affects almost half of all adults in rural South Africa [3,7], meaning that the majority of adults in rural South Africa are therefore living with HIV, hypertension, or both. For these individuals, achieving disease control and maintaining health requires early and sustained interaction with the health care system. Primary health care (PHC) services must be of high quality to engage and retain individuals over time while managing these lifelong conditions [8–10].

Individual factors contribute to treatment retention, with evidence that retention in HIV treatment is lower for men, younger adults, and those recently initiating treatment [11–16]. For men and younger adults in South Africa, these patterns may reflect distance or inconvenience in reaching health services, wariness of public health services due to concerns of stigma or judgmental care, and frequent migration for schooling or employment opportunities. While there is less evidence to date on retention in hypertension treatment in South Africa, a recent review found that men, the elderly, and those of lower socioeconomic status or lower health literacy were less likely to remain in care for either HIV or hypertension [17].

Over the past decade, the government of South Africa has implemented an Integrated Chronic Disease Management model explicitly to strengthen primary care and leverage past investment in priority areas like HIV services to strengthen NCD services in order to better support and retain all patients [18]. Implementation has been stymied in rural areas by inadequate resources (staff, supplies, medication) [3,19]. Poor patient experience such as breaches in confidentiality, long wait times and disrespectful care providers undermine individuals’ confidence in health services and willingness to remain in treatment [20,21]. The impacts of insufficient quality of care on population health are substantial: estimates from rural settings in Mpumalanga province suggest less than 30% of people living with HIV are diagnosed, on ART, and virally suppressed, while an estimated 20% of those with hypertension are diagnosed, in treatment, and achieving blood pressure control [22,23]. The situation worsened with the profound disruption during the coronavirus disease 2019 (COVID-19) pandemic: routine services and non-COVID hospitalisations declined dramatically in during the initial level five alert (movement for essential goods and services only) from 26 March to 30 April, with early indications of increased morbidity and mortality as restrictions were eased [24,25]. Clinic operations continued to be affected throughout the pandemic period by varying levels of restrictions on gatherings such as in-person staff meetings, by exposure and illness among health care personnel, and by resource allocation towards the pandemic and subsequent vaccination campaigns [26].

Addressing the long-standing challenges in health service provision and ensuring resilient health systems to withstand crises demands innovative approaches to health system strengthening [27–29]. Existing interventions show limited effectiveness in changing patient outcomes [30]. For example, one of the largest such programmes in South Africa, the national Ideal Clinic programme, demonstrated increases in inputs to care (e.g. provider training, guideline availability, essential equipment and medications) but no improvement in HIV care outcomes [31]. An analysis of quality of HIV care based on longitudinal laboratory data found that 52% of variation in quality is explained at the facility level [32]. Similarly, randomised controlled trials for HIV and hypertension interventions have identified substantial variation in clinic implementation as a key barrier to intervention success [3,33]. While resources such as staffing and supplies are important in shaping clinical care, this evidence suggests they are far from sufficient in explaining observed variation. Clinic-level organisational factors are promising – albeit complex – targets for interventions to produce sustained improvements across multiple services [34].

Frameworks developed in the United States posit key constructs such as organisational capacity to implement change as determinants of implementation success and ultimately improved patient outcomes [35,36]. Organisational research in the South African health system has identified the importance of effective leadership in addressing stressful circumstances and building a culture of teamwork for ongoing improvement [37,38]. In-depth qualitative research within PHC clinics attests to the critical role of leadership skills among managers to navigate competing demands and resources constraints and to cultivate teamwork and problem solving among providers [39–42]. However, quantitative evidence regarding the impact of clinic factors on patient outcomes is lacking. In this study, we test measures of leadership, cohesion, and stress within PHC clinics and assess whether clinic-level measures are associated with patient retention for chronic conditions of HIV and hypertension both before and during the COVID-19 pandemic.

## METHODS

### Study setting

This study took place in the rural Bushbuckridge subdistrict of Mpumalanga province in northeastern South Africa, within the area covered by the Agincourt Health and Socio-Demographic Surveillance System (HDSS) [43]. The sampling frame of the HDSS, which has been active since 1992, covers over 20 000 households and 115 000 resident individuals within 420 km^2^ [33,44]. Over 20% of adults are living with HIV and >50% of adults over 40 have elevated blood pressure [45,46]. Nine PHC facilities (smaller clinics and larger community health centres) are located in or adjacent to the area covered by the surveillance system.

### Data sources

We conducted a two-part clinic quality assessment in the nine PHC facilities serving the villages covered by the Agincourt-HDSS. The assessment included an audit of clinic infrastructure conducted from June to August 2018 and interviews with providers from January to March 2019 [47–50]. We drew a convenience sample based on availability of nurses and lay counsellors during two to three days of provider interviews in each facility. We targeted five respondents per smaller clinic (out of 8–16 total nurses and 1–2 lay counsellors) and eight in the larger health centres staffed by 24–41 nurses and 4–7 lay counsellors. Study procedures are described in full elsewhere [48].

As part of the ongoing research within the Agincourt HDSS, the HDSS-Clinic Link electronic clinical tracking system was established in 2014 to capture longitudinal clinical data from consenting adults aged 18 and older engaged in chronic care (HIV and NCD) in PHC facilities [51]. Following participant consent, clinic visit information such as visit date, diagnoses, and prescriptions are captured from individual paper-based records into an electronic system. Record linkage is conducted to match clinic patients to their corresponding Agincourt HDSS record [51]. While the HDSS-Clinic Link was active in the nine facilities included in the quality assessment as of late 2018, it was subsequently deactivated in two facilities, leaving seven facilities with clinic quality assessment data and complete patient records for this study period. Data entry paused from 26 March to 30 April 2020, but clinical staff maintained patient files for any visits during this time; these visits and subsequent visits were entered into the HDSS-Clinic Link after data entry resumed once the alert level was reduced.

For the purpose of this study, we identified all individuals aged 18 and over with a diagnosis of HIV and/or hypertension active in these seven clinics between 1 January 2019 and 31 December 2021, including those with treatment regimens for HIV or hypertension if no diagnosis was documented. We excluded women with recent pregnancy diagnosed with hypertension to avoid combining pregnancy-induced hypertension cases with the condition of interest, chronic hypertension. Pregnancy-induced hypertension differs from chronic hypertension in its incidence, treatment, and outcomes; it is managed within maternal care services and typically resolves post-partum [52,53].

### Measures

We used two measures from the facility audit to characterise clinics: nursing capacity, defined as the number of nurses (professional nurse and enrolled nurse) assigned to the facility, and service readiness index, an average score of inputs to care in domains of infrastructure (amenities), medication, equipment, and supplies based on WHO standards and adapted to focus on HIV care at clinics [48,54].

To quantify the constructs of leadership, teamwork, and stress identified in qualitative research in this setting, we drew on scales validated in high-income settings and in a South African community setting (Table S1 in the **Online Supplementary Document**). We used four-item scales for leadership engagement and stress developed in health facilities in the USA [55]. Example items are: ‘Leadership in this clinic creates an environment where things can be accomplished’ and ‘I am under too many pressures to do my job well’ [55]. We adapted a community social cohesion scale validated locally [56] into a five-item measure to capture teamwork within the clinic (e.g. ‘Clinic staff members work together as a team’). While not previously validated in the study clinics, these three measures and comparable items were also used in a subsequent study in the neighbouring province of KwaZulu-Natal, which included expert panel confirmation and cognitive interviews to assess content validity [57]. Agreement with each statement was captured with a five-point Likert scale from strongly disagree = 1 to strongly agree = 5. Leadership engagement items were all positively worded; one negatively worded cohesion item was reverse coded so that higher scores represented stronger cohesion for all items. Stress items were all worded such that agreement indicated higher stress.

To describe the patient population, we extracted demographics of age, sex, and village of residence. We categorised age as 18–29 years old, 30–39, 40–49, 50–59, 60–69, and 70 and over. Clinical indicators included time since diagnosis for those with HIV; time since diagnosis of hypertension was not available in the Clinic Link data. We identified patients enrolled in Central Chronic Medicines Dispensing and Distribution (CCMDD) programme, which provides longer duration of prescriptions with fast-track or external pick up of prescription refills for those with stable disease status. Implementation of CCMDD was slow and varied between clinics prior to the COVID-19 pandemic [48], but accelerated as guidelines for eligibility were loosened during the pandemic. We classified patients no longer in care based on documented transfer out of the treating clinic and confirmed mortality from the Agincourt HDSS census data. Mortality data could be obtained only for those patients with a confirmed matching record in the Agincourt HDSS database.

We defined loss to follow up based on the South African National Department of Health (NDoH) guidelines as failure to return to clinic within 90 days of either the next scheduled visit or end of active prescription in the absence of a visit date. This captures treatment interruption as well as sustained loss to follow-up. For records where both scheduled visit date and prescription duration were missing, we imputed the patient’s modal visit interval to calculate expected next visit date. We created a data set for analysis with one observation per patient per quarter active in care. Patients were considered eligible for all quarters within the study period following treatment initiation until transfer out, death, or end of the study period. The outcome for each eligible quarter was a binary indicator of retained in care or lost (missing a visit scheduled in that quarter or a prescription end date in the quarter and not returning within 90 days). Patients in treatment for both HIV and hypertension were classified as retained if there was evidence of a visit or medication refill for either condition. Analytic data were stratified into the period before COVID-19 (January 2019–March 2020) and during COVID-19 (April 2020–December 2021). Individuals permanently lost to follow up before April 2020 were not included in the COVD-19 period.

### Data analysis

We used descriptive statistics to report median and interquartile range (IQR) for nursing capacity and service readiness. We calculated Cronbach’s alpha to assess inter-item agreement for each scale. We calculated a_wg_ as a metric of within-facility agreement considering each provider and item as a rating of the facility leadership, cohesion, or stress. a_wg_ ranges from −1 to 1 and can be interpreted akin to Cohen’s kappa, with scores of 0.5 and higher indicating at least moderate agreement between raters [58]. Finally, we used providers’ average agreement with each scale as an individual summary measure and calculated the intraclass correlation coefficient (ICC) to quantify within vs between facility variance, interpreting ICC>0.05 as evidence of minimum within-facility reliability. We assessed correlation between each clinic-level measure using *P* < 0.050 as evidence of significant correlation.

We used descriptive statistics to summarise the characteristics of the eligible patients in treatment for HIV and/or hypertension. We plotted retention by quarter for each condition by facility as well as by demographic and clinical characteristics. We used generalised estimating equation (GEE) models with a Poisson link and robust standard errors to analyse the repeated measures of quarterly retention within person. In addition to accounting for correlation between repeated measures on the same individual, GEE models provide population-average estimates; in this case, the expected difference in retention rate if all clinics had a given level of an exposure like leadership [59]. We tested exchangeable and first order auto-regressive correlation structures and selected the correlation that minimised the quasi-likelihood under the independence model criterion. We tested age, sex, and multi-morbidity of HIV and hypertension in unadjusted models stratified by condition to understand demographic and clinical predictors of retention. To assess the role of clinic factors in influencing retention before and during the COVID-19 pandemic, we used the GEE models to regress individual retention on clinic inputs – nursing capacity and service readiness − and each organisational measure: leadership, cohesion, and stress. We identified individual characteristics known to affect retention, such as age, sex, distance from facility, and need for additional services. While individual characteristics would not cause differences between clinics, these same characteristics could shape choice of facility; we adjusted all models for the case mix variables of age, sex, village of residence, and comorbidity of HIV and hypertension. We controlled for time (quarters from period start) in each model to account for temporal trends. We conducted analyses stratified by disease outcomes and separately for the pre-pandemic and pandemic periods.

### Ethical review

Data for this project were derived from completed studies at the Agincourt HDSS research site that were previously approved by the Institutional Review Boards at the University of California San Francisco and the University of Witwatersrand.

## RESULTS

The nine facilities assessed had a median of 12 nurses on staff (IQR = 9–17) and a score of 0.83 out of 1 on the input to care index (IQR = 0.78–0.89). The 2019 clinic quality assessment included 54 participants from these nine facilities, including 52 of the total 132 nurses (39%) employed at these facilities.

All providers approached to participate consented and completed the assessment. Agreement was highest with cohesion items (average 4.3 out of 5), followed by stress and leadership (**Table 1**), indicating an environment in which providers felt part of a team, experienced stress in their roles, and rated clinic leadership moderately positively. All three measures showed high inter-item correlation and moderate to strong intra-facility agreement, particularly cohesion. All facilities showed at least moderate agreement on cohesion items, and 50% of total variance in responses was explained by the facility. We found no statistically significant correlation between measures of facility inputs and organisational factors (data not shown).

**Table 1:** Level and scale performance of measures of leadership, cohesion, and stress within clinics, January–March 2019

Variables	Provider average ± SD (N = 54)	Facility average ± SD (N = 9)	Intra-item correlation (Cronbach’s alpha)	Median a_wg_* (N clinics with a_wg_≥0.5)	ICC
Leadership	3.63 ± 1.24	3.69 ± 0.87	0.94	0.52 (6)	0.38
Cohesion	4.30 ± 0.54	4.25 ± 0.43	0.74	0.92 (9)	0.50
Stress	3.93 ± 0.97	3.96 ± 0.55	0.87	0.64 (5)	0.16

ICC – intraclass correlation coefficient, SD – standard deviation

*a_wg_ is a measure of within group agreement that ranges from −1 to 1, with 0.5 used as a standard threshold for moderate agreement; it can be interpreted as an analogue to Cohen’s kappa at the group level.

After excluding 133 women with hypertension diagnoses and recent pregnancy, and 21 incomplete records, a total of 19 445 individuals met eligibility as enrolled in HIV and/or hypertension treatment during the 2019–2021 study period, contributing a total of 194 369 person-quarters of time from individual quarter of entry to the end of the study period in December 2021. Nearly twice as many individuals were in treatment for HIV as for hypertension, despite the higher population prevalence of hypertension. Over the course of the study, 623 individuals (3.2%) were documented as transferring out of a study facility and 482 were confirmed as deceased (2.5%).

Women made up the three quarters of patients for each condition (**Table 2**). As expected, based on disease incidence, the majority of individuals receiving HIV treatment were below 50 years old, while the reverse was true for hypertension treatment. Multimorbidity was common: 2678 individuals were in treatment for both HIV and hypertension, making up 18% of HIV patients and 36% of hypertension patients. The median patient in HIV treatment was diagnosed nearly five years prior and initiated ART four years prior. Close to half of HIV patients and one third of hypertension patients had ever been enrolled in the CCMDD programme to facilitate chronic condition management through fast-track dispensing or external prescription pick-ups. Distribution of patients across the seven study PHC facilities was somewhat different between the two conditions, although for both conditions, two facilities (1 and 6) treated a plurality of patients.

**Table 2:** Characteristics of individuals in HIV and hypertension treatment programmes across seven PHC facilities in Agincourt HDSS study area, 2019–2021

Variables	Individuals receiving treatment for HIV (N = 14 693)	Individuals receiving treatment for hypertension (N = 7429)
**Sex**		
Female	10 961 (74.6%)	5560 (74.8%)
Male	3732 (25.4%)	1869 (25.2%)
**Age group**		
18–29	2882 (19.6%)	165 (2.2%)
30–39	4577 (31.2%)	707 (9.5%)
40–49	3744 (25.5%)	1343 (18.1%)
50–59	2108 (14.3%)	1706 (23.0%)
60–69	1012 (6.9%)	1650 (22.2%)
70 and over	370 (2.5%)	1858 (25.0%)
**HIV and hypertension comorbidity**		
No	12 016 (81.8%)	4752 (64.0%)
Yes	2677 (18.2%)	2677 (36.0%)
**Years since HIV diagnosis**		
Median (Q1, Q3)	4.75 (1.65, 7.85)	NA
**Years since ART initiation**		
(Q1, Q3)	4.07 (1.39, 7.31)	NA
**Ever on CCMDD**		
No	8152 (55.5%)	4846 (65.2%)
Yes	6541 (44.5%)	2583 (34.8%)
**Health facility**		
Facility 1	2990 (20.3%)	1271 (17.1%)
Facility 2	1636 (11.1%)	586 (7.9%)
Facility 3	1711 (11.6%)	1131 (15.2%)
Facility 4	1077 (7.3%)	478 (6.4%)
Facility 5	1933 (13.2%)	1444 (19.4%)
Facility 6	4521 (30.8%)	1796 (24.2%)
Facility 7	825 (5.6%)	723 (9.7%)

ART – Antiretroviral Therapy, CCMDD – Central Chronic Medicines Dispensing and Distribution, HDSS – Health and Socio-Demographic Surveillance System, HIV – human immunodeficiency virus, PHC – primary health care, Q – quarter

**Figure 1:**
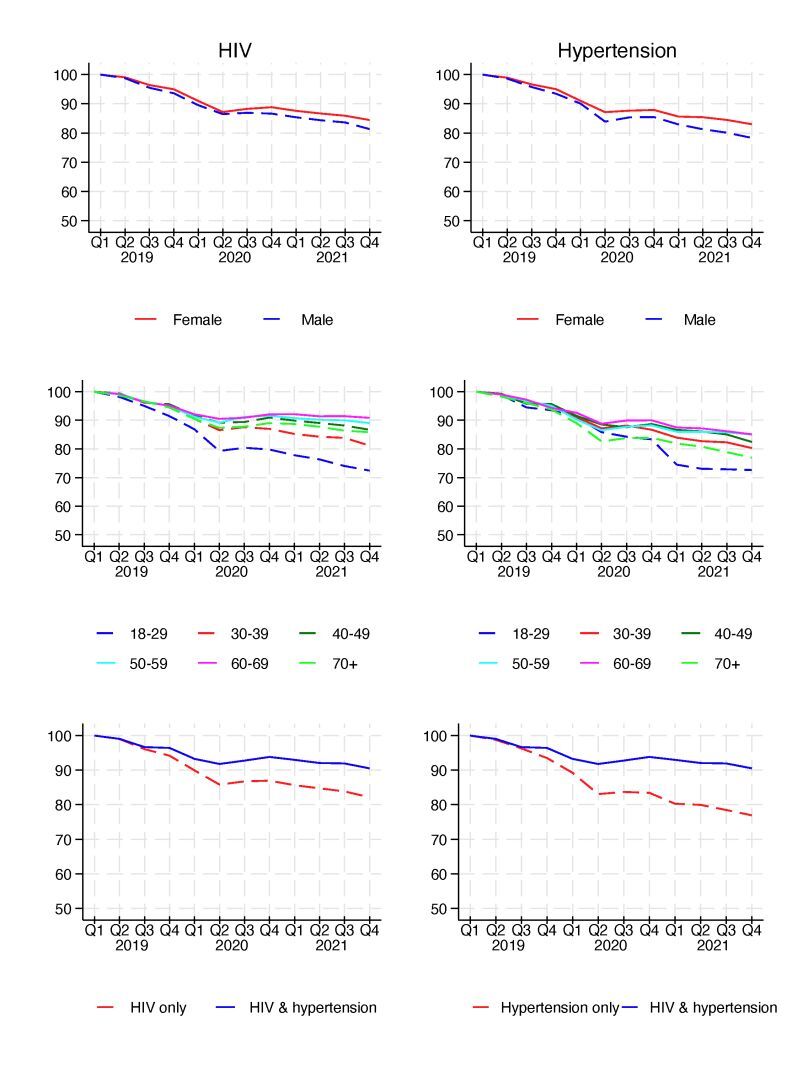
Retention in chronic care across seven PHC facilities in Agincourt HDSS study area before and during the COVID-19 pandemic. Percent of patients retained in care is shown for each condition, overall and by facility; the y-axis spans 50 to 100%. Dashed lines indicate facilities with statistically significant difference in retention from reference facility (Facility 6, largest patient volume) at *P* < 0.050. The start of the COVID-19 pandemic is shown in the vertical red line. HDSS – Health and Socio-Demographic Surveillance System, PHC – primary health care

The cohort of patients active in HIV treatment experienced a few percentages point loss per quarter, indicating that within our open cohort from 2019 to 2021, more patients left clinics or were otherwise lost to follow up than initiated treatment. Retention declined to 88% as of first quarter of 2020, remaining close to that figure for the rest of 2020, and then fell to 84% by the end of 2021 (**Figure 1**). Retention was comparable for patients receiving treatment for hypertension, with 89% retained as of first quarter of 2020 and 82% at the end of the study period. For both conditions, the second and third quarter of 2020 at the start of the COVID-19 pandemic saw the least drop out of any study period. Retention varied modestly between clinics, with differences up to 11% between highest and lowest retention for HIV treatment (second quarter of 2020) and up to 15% between highest and lowest for hypertension treatment (second quarter of 2020 and third quarter of 2021). Relative performance was consistent across conditions, with facilities tending to have similarly above or particularly below average retention for both HIV and hypertension treatment.

**Figure 2:**
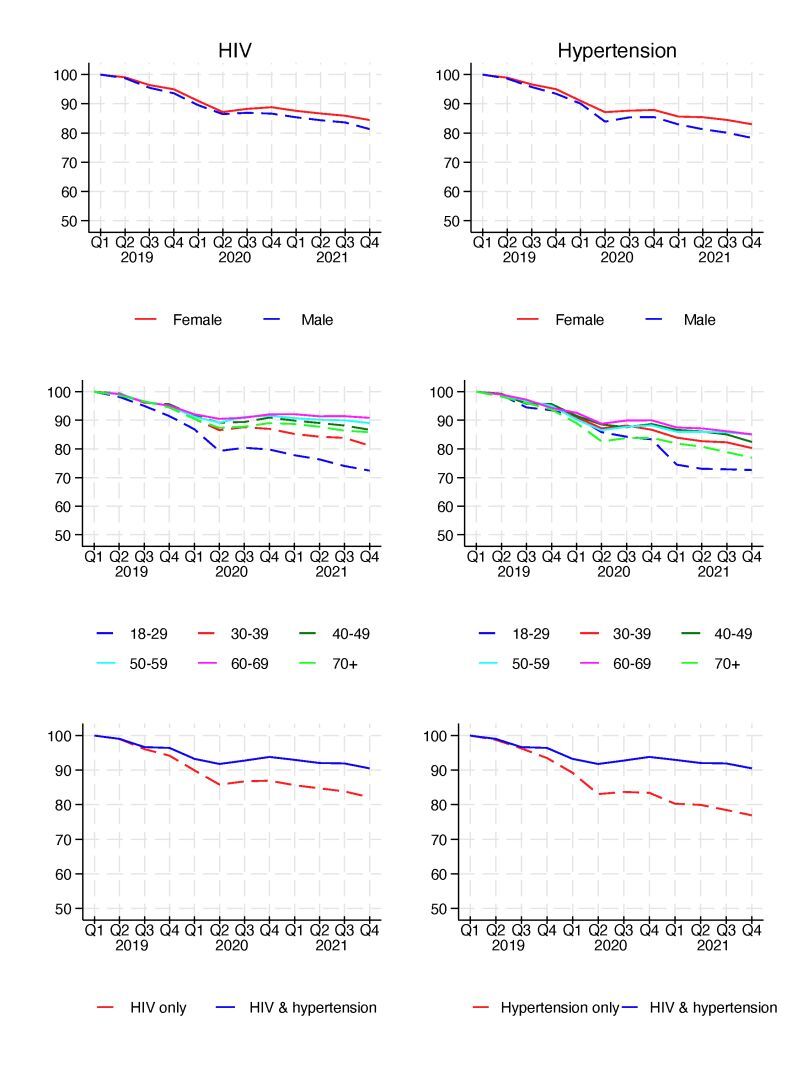
Retention in care by demographic and clinical characteristics in Agincourt HDSS study area. Percent of patients retained in care is shown for each condition, overall and by age, sex, and comorbidity. Dashed lines indicate retention significantly lower than the reference (most highly retained group) at *P* < 0.050 using unadjusted GEE Poisson models with autoregressive correlation structure. HDSS – Health and Socio-Demographic Surveillance System

Demographic and clinical predictors of retention were also similar across conditions (**Figure 2**), with men less likely to be retained than women and both younger (18–29) and older (≥70-year-old) adults less likely to be retained than those 60–69 years old. For HIV alone, working aged adults 30–39 and 40–49 also experienced lower retention than the reference category of 60–69-year-olds. Being in treatment for both conditions was associated with much higher retention (90.5% compared to 82.1% of those in treatment for HIV alone and 76.9% of those in treatment for hypertension alone).

Model fitting supported use of a first order auto-regressive correlation structure for all models of clinic factors as predictors of retention. Two hundred and seven individuals in HIV treatment and 100 individuals in hypertension treatment were permanently lost to follow-up prior to the start of the COVID-19 pandemic; they were removed from the second portion of the analysis.

Clinics with more nursing capacity and greater readiness for services did not tend to have higher retention – in fact, prior to the COVID-19 pandemic, the only statistically significant association of these factors was greater service readiness associated with lower retention in HIV treatment (**Table 3**). During the pandemic, greater service readiness was linked to higher retention for HIV treatment, but lower retention in hypertension treatment.

**Table 3:** Association of clinic inputs and organisational factors with retention in chronic disease care before and during the COVID-19 pandemic in Agincourt HDSS study area

A: Before COVID-19, 2019–first quarter 2020		
	HIV treatment (N = 49 366 person-quarters)* IRR (95% CI)	Hypertension (N = 26 488 person-quarters)* IRR (95% CI)
Nursing capacity	1.00 (1.00–1.00)	1.00 (1.00–1.00)
Service readiness	0.94 (0.90–0.97)†	0.97 (0.91–1.02)
Leadership	1.00 (1.00–1.01)	0.99 (0.98–0.99) †
Cohesion	0.99 (0.98–0.99)†	0.99 (0.98–1.01)
Stress	1.02 (1.01–1.03)†	1.01 (0.99–1.02)
**B: During COVID-19, second quarter 2020–end 2021**		
	HIV treatment (N = 87 721 person-quarters)* IRR (95% CI)	Hypertension (N = 45 564 person-quarters)* IRR (95% CI)
Nursing capacity	1.00 (1.00–1.00)	0.99 (0.99–1.00)
Service readiness	1.06 (1.00–1.13)†	0.90 (0.80–0.99)†
Leadership	0.98 (0.97–0.99)†	0.96 (0.95–0.98)†
Cohesion	1.02 (1.00–1.03)†	1.01 (0.98–1.04)
Stress	0.99 (0.98–1.01)	0.97 (0.94–1.00)†

CI – confidence interval, COVID-19 – coronavirus disease 2019, HDSS – Health and Socio-Demographic Surveillance System, IRR – incidence rate ratio.

*Models control for factors that may be associated with retention and that could affect patient case mix by facility: age group, sex, village, and co-morbidity. We also controlled for time. Each clinic factor is modelled individually.

†Estimates are significant at *P* < 0.050.

In examining associations between organisational factors as measured in 2019 and retention in treatment from 2019 to 2021, we found limited evidence to support our hypotheses. Associations of clinic measures of leadership, cohesion, and stress with retention in HIV treatment and hypertension treatment were weak. Prior to the pandemic period, lower cohesion and higher stress were linked to higher retention in HIV treatment; higher leadership scores were linked to lower retention in hypertension treatment. After the pandemic, leadership was linked to lower retention in treatment for each condition (**Table 3**). We did find that cohesion was associated with better retention in HIV treatment (incidence rate ratio (IRR) = 1.02; 95% confidence interval (CI) = 1.00–1.03) and that higher stress was associated with lower retention in hypertension treatment (IRR = 0.97; 95% CI = 0.94–1.00).

## DISCUSSION

This study demonstrated that measures of leadership, cohesion, and stress were generally reliable within individual respondents and at the facility level. Capacity to retain patients in treatment programmes did vary between facilities, up to 11% for HIV and 15% for hypertension over this two-year period; the same facilities had below average retention for both conditions. Retention was highest for individuals with both conditions, followed by those with HIV alone and lowest for those with hypertension alone, underscoring the gaps in hypertension disease management despite the increasing health burden. Clinic staffing was not linked to retention outcomes, while service readiness showed inconsistent associations with retention. We found limited evidence supporting a hypothesised association of more positive clinic organisational factors with retention in care. Higher cohesion was linked to retention for HIV and lower stress to retention for hypertension treatment during the COVID-19 pandemic period, but given that we found reverse or null associations pre-pandemic, evidence is at best inconclusive.

Our analysis of retention in treatment found over 80% retention at two years for HIV and hypertension, with sustained retention during the COVID-19 pandemic. This suggests that despite the initial sharp drop of clinic visits, the increase in CCMDD enrolment and eventual return to clinic visits protected against substantial pandemic-related treatment attrition of over 90 days. Clinics and patients together were largely able to maintain essential treatment within the constraints of the pandemic response. These findings align with evidence from KwaZulu-Natal that increased duration of ART prescriptions within CCMDD adopted in response to the pandemic supported continued retention on treatment and sustained disease control [60]. Particularly if health benefits are similar for patients with hypertension in CCMDD, consistent implementation of CCMDD programmes with longer dispensing times could provide substantial patient benefit [61].

Overall, we found that men and younger adults were less likely to be consistently retained on treatment, in keeping with prior evidence [12–16,62]. We also found that adults over 70 experienced lower retention, potentially due to mortality not recorded in clinic records. Efforts to identify those truly lost from treatment – vs undocumented transfer, outmigration, or mortality – are warranted. Individuals with comorbidity were substantially more likely to remain in care, expanding on prior studies attesting to improved access to and high retention in NCD care among adults in treatment for HIV [46,63,64]. Retention was lowest for those with hypertension alone, and the overall population in treatment for hypertension was half the number of those in treatment for HIV despite high population prevalence of both conditions [45,63,65,66]. While indicators of hypertension detection and management have improved in this area in the years preceding this study [67], this evidence indicates continued deficits relative to the long-standing HIV care programme.

There are multiple explanations possible for our inconsistent findings around clinic factors and patient retention. Facilities with higher service readiness tended to be more centrally located, with larger patient populations and longer opening hours; they may have been accessed by patients living farther away who later transferred to smaller, closer facilities. We measured leadership, cohesion, and stress within clinics at a single point in time in early 2019, and these factors could have changed over the course of the study, particularly with turnover of providers or clinic leaders before and during the pandemic. For instance, leadership engagement would be expected to change with leadership transitions. Measuring these factors over time would enhance understanding of the variability of these conditions and improve estimation of their contributions to patient outcomes. It is also possible that the aspects of clinic organisation as measured in this study are not strongly predictive of better patient management and retention, or at least not to a degree that can be detected within seven PHC facilities in the same municipality and subject to the same resource constraints and external shocks. We did observe notable differences in retention between facilities, including declines across both conditions in two facilities in late 2019 and early 2020 that are not easily explained by external factors. This underscores the need to monitor facility-level performance in patient retention across conditions to support earlier identification and response to such shortfalls. In addition, since many adherence and retention interventions focus largely on supporting individuals [15,68–70], the differences we identified in retention between facilities highlights the need for increased understanding of modifiable clinic-level factors.

Strengths of this study include the in-depth quantitative assessment of clinic leadership, cohesion, and stress as well as the use of a large clinical data set with longitudinal patient data across health conditions. Study findings are limited by several factors. We were not able to perform a separate validation study of the scales prior to the quality assessment to confirm validity in the study clinics. Clinic patients from outside the surveillance area or with incomplete demographic data could not be linked to the Agincourt HDSS mortality data. Documentation of patient transfer to local or further facilities is known to be far from complete [71,72]. These factors introduce error in our retention estimates due to incomplete censoring of those who were actually deceased or enrolled at a different facility. HDSS-Clinic Link records did not include clinical history of hypertension.

## CONCLUSIONS

Retention in treatment of HIV and for hypertension remained stable in the second and third quarters of 2020 during the COVID-19 pandemic in this rural setting, particularly for those with both conditions. These findings may be generalisable to other rural areas in South Africa such as KwaZulu-Natal and in southern African countries with similar provision of government health care and expanded treatment pick up programmes during the pandemic; they may be less relevant in urban areas, where patients have access to more public facilities as well as private sources of care, or in settings dependent on in-person medication refills. Further efforts to bolster retention among men and younger adults, including sustaining the expanded criteria for CCMDD enrolment, are warranted. While clinic factors may prove important in implementation of new programmes, this study provides little conclusive evidence of their role in ongoing treatment success.

## Additional material


Online Supplementary Document
