# Supplementary material for: Leadership, cohesion, and stress in primary care facilities and retention in chronic care in rural northeast South Africa before and during the COVID-19 pandemic: A longitudinal study
Source: J Glob Health. 2024 Dec 9;14:05035. doi: 10.7189/jogh.14.05035 (PMC11627195; doi:10.7189/jogh.14.05035)
Supplement: Online Supplementary Document [file jogh-14-05035-s001.pdf]

Table S1: Scale Items for Leadership, Teamwork, and Stress

Now we will read some general statements about this facility. The following questions refer to the people who work at this health facility, including yourself. Response options are strongly disagree, somewhat disagree, neither agree nor disagree, somewhat agree, strongly agree.
**Leadership**
The clinic leadership makes sure that we have the time and space necessary to discuss changes to improve care.
Leadership in this clinic creates an environment where things can be accomplished.
Clinic leadership promotes an environment that is an enjoyable place to work.
Leadership strongly supports clinic change efforts.
**Cohesion (Teamwork)**
Clinic staff are usually quick to help one another when needed.
Mutual trust among clinic staff is strong
There is often tension among people who work at this clinic.
Clinic staff members work together as a team.
People who work in this clinic share similar goals for the clinic.
**Stress**
I am under too many pressures to do my job well.
Staff members often show signs of stress and strain.
The heavy workload here reduces the quality of care.
Staff frustration is common here.
